# Clinical performance of immediately placed and restored progressive-type implants in the esthetic zone: a prospective observational study

**DOI:** 10.1186/s40729-022-00462-y

**Published:** 2022-11-22

**Authors:** G. Trimpou, F. Schwarz, A. Begić, P. Hess, J. Lermen, N. Keim, K. Obreja, P. Parvini

**Affiliations:** Department of Oral Surgery and Implantology, Goethe University, Carolinum, Frankfurt, Germany

**Keywords:** Clinical study, Immediate implant placement, Immediate restoration

## Abstract

**Purpose:**

To assess implant success and survival of immediately placed and restored progressive-type implants in the esthetic zone.

**Material and methods:**

A total of *n* = 21 patients (21 implants) had received an immediate placement of a tapered, two-part implant with a progressive thread design (PL) for a single tooth replacement in the anterior maxilla. An immediate ‘non full-functional loaded’ restoration was provided upon adequate primary stability on a final patient-specific abutment (one abutment-one time concept). The final restoration was provided at 12 weeks (baseline). Implant survival and success (e.g. bleeding on probing—BOP, probing pocket depth—PD, mucosal recession—MR, pink esthetic score—PES) as well as patient- reported outcomes (PROM`S) were recorded at 6 and 12 months.

**Results:**

An adequate primary implant stability (i.e. insertion torque > 35 Ncm) was obtained at all but one sites. At 12 months, implant survival (*n* = 20 patients) amounted to 100%. Non-significant changes to baseline were noted for mean BOP (2.5 ± 28.2%), PD (− 0.26 ± 0.73 mm), and MR (0.0 ± 0.4 mm) values. PES values amounted to 12.9 ± 1.14 and 13.2 ± 0.84 at 6 and 12 months. Technical and mechanical complications were not observed. Patients expressed an overall high satisfaction.

**Conclusions:**

The presented immediacy protocol was associated with high survival and success rates on the short-term.

**Graphical Abstract:**

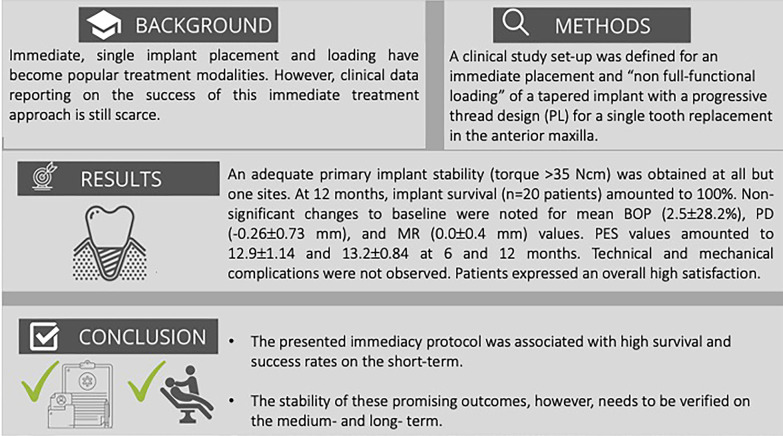

## Introduction

Nowadays, immediate implant placement has evolved to an evidence- based intervention for single-tooth replacements [1]. Its efficacy was proven to be on a level equivalent to that noted for a conventional, delayed implant placement, as indicated by a comparable implant survival (94.9% vs. 98.9%), as well as clinical (i.e. probing depths) and esthetic outcomes noted between 12 and 96 months [2]. Even though the underlying evidence is very limited, immediate provisionalization did not seem to have an influence on the survival and success rates [3]. Apart from a decreased treatment time and avoidance of a removable provisional prosthesis [4], the mechanical stimulus associated with a controlled immediate restoration may also contribute to the quality and quantity of bone formation [5]. On the contrary, however, immediate implant placement and the achievement of an adequate primary stability may clearly be challenged by the integrity and morphology of the extraction socket [6]. The initial mechanical implant stability is of particular relevance when an immediate restoration protocol is considered. Accordingly, for the latter scenario, insertion torques of ≥ 20 to 45 Ncm have commonly been recommended [7], even though optimal torque values to support the secondary implant integration are yet to be determined. To overcome some of these potential limitations, the thread designs and external geometries have undergone several modifications [8], particularly to enhance the primary implant stability at extraction sockets. A recently introduced progressive-type two-piece implant (PL) combines different thread designs spanning from the upper parallel segment down to the apex of the lower tapered segment. In vitro data have indicated that this particular implant design was associated with adequate primary stability values in a very challenging type-4 bone model [9], thus suggesting its suitability for immediate protocols.

Therefore, the present prospective observational study aimed at evaluating implant survival and success of immediately placed and restored PL implants for single-tooth replacements in the esthetic zone.

## Material and methods

### Study design and participants

A total of 21 patients, each exhibiting one non-retainable tooth in the anterior maxilla attended the Department of Oral Surgery and Implantology at the Goethe University Frankfurt, Germany. All patients had received an immediate type-1 placement [1] of a tapered, two-part implant with a progressive thread design (PL) for a single tooth replacement in the anterior maxilla (014–024 FDI). The patient- and implant site characteristics as well as reasons for tooth extraction are presented in Tables 1 and 2. All surgical procedures were carried out between March 2019 and May 2021.

**Table 1:** Patient characteristics

Patient age	48.0 ± 13.7 years; range 21 to 67 years
Female/male	*n* = 11/9
ASA physical status classes	I (normal healthy) = 15 II (mild systemic disease) = 5
Smoking	None = 14 < 10 cigarettes per day = 6

**Table 2:** Reasons for tooth extraction and implant site characteristics

Reason for tooth extraction	Caries = 2 Endodontic lesion = 6 Fracture = 11 Persistent deciduous tooth = 1
Defect dimension	Width = 1.33 ± 2.69 mm Length = 3.11 ± 6.19 mm
Mucosal thickness	1.23 ± 0.45 mm
Distance implant—adjacent tooth	Mesial = 2.44 ± 0.72 mm distal = 2.27 ± 0.56 mm
Implant diameters	3.3 mm = 3 3.8 mm = 7 4.3 mm = 8 5.0 mm = 2
Implant length	11 mm = 2 13 mm = 13 16 mm = 5
Insertion Torque	36.66 ± 7.07 Ncm

An immediate ‘non full-functional loaded’ restoration was provided upon adequate primary stability (insertion torque > 35 Ncm) on a final patient-specific abutment (one abutment-one time concept). The final restoration was provided at 12 weeks.

The primary outcomes included implant survival and success (bleeding on probing—BOP, probing pocket depth—PD, mucosal recession—MR) at 12 months. The secondary outcomes included plaque index—PI, pink esthetic score—PES, and patient-reported outcome measures (PROM’S).

The study protocol was approved by the ethics committee of the Goethe University, Frankfurt, Germany and registered via the Internet Portal of the German Clinical Trials Register (DRKS00016500). Each patient was given a detailed description of the study procedures and signed an informed consent before participation. The present reporting considered the checklist items as proposed in the STROBE statement [10].

### Sample size calculation

Due to a lack of reference data on a similar immediacy protocol in the literature, a sample size calculation was not deemed feasible. Due to the COVID-19 pandemic, the number of included patients had to be limited to *n* = 21 (originally planned: *n* = 25).

### Inclusion and exclusion criteria

Patients were initially included in the study if they presented all of the following conditions: (1) males and females with an age of 18–65 years, (2) planned single tooth replacement in the maxilla (14–24 FDI) allowing the placement of a single implant, (3) adjacent teeth have to be free of acute or chronical infections (e.g. periodontal or endodontic problems), (4) single tooth gaps (i.e. natural tooth roots present on the sides adjacent to the implant), (5) opposing dentition must be natural teeth or fixed restoration, (6) subject shall have a stable occlusal relationship, (7) patient has been informed of the follow-up visits and is willing to return to the clinical center for these follow-up visits, (8) subjects must have voluntarily signed the informed consent form before any study related action.

Exclusion criteria included: (1) Systemic diseases that would interfere with implant therapy (e.g. uncontrolled diabetes (HbA1c > 7%), (2) general contraindications for dental and/or surgical treatments, (3) patients who smoke > 10 cigarettes per day or cigar equivalents, or who chew tobacco, (4) bone metabolism disorders, (5) uncontrolled para-functional diseases (bruxism, clenching or grinding of teeth), (6) disorders that impede the ability of patients to maintain adequate oral hygiene, (7) conditions or circumstances, in the opinion of the investigator, which would prevent completion of study participation or interfere with analysis of study results, such as history of non-compliance or unreliability, (8) pregnant or breastfeeding women.

### Surgical procedures

All patients had received a perioperative single- shot antibiotic medication (Amoxicillin 2 g) and were treated under local anesthesia (4% articaine plus epinephrine 1:200,000). The flapless atraumatic extraction of the respective teeth was accomplished using forceps or the Benex® (Helmut Zepf Medizintechnik GmbH, Seitingen-Oberflacht, Germany) system. This was followed by a careful granulation tissue removal and inspection of the integrity of the socket walls using a periodontal probe. Implant site preparation was performed according to the surgical protocol recommended by the manufacturer. Platform-switched PL implants (Camlog Progressive Line®, Camlog, Wimsheim, Germany) were placed in a subcrestal position (2–3 mm) along the palatal wall of the extracted socket to ensure a distance of 1.5–2 mm to the line connecting the emergence point of the adjacent teeth (i.e. comfort zone) [11]. The primary stability was assessed by insertion torque measurements (Implantmed, W&H, Bad Reichenhall, Germany). Peri-implant gap grafting was accomplished using a natural bone mineral (BioOss spongiosa, Geistlich Biomaterials, Wolhusen, Switzerland).

### Prosthetic procedure

Before surgery, conventional impressions were taken with polyether (Impregum NF, 3M ESPE, Neuss, Germany) to produce master casts (SheraJive, Shera, Lemförde, Germany).

**Fig. 1:**
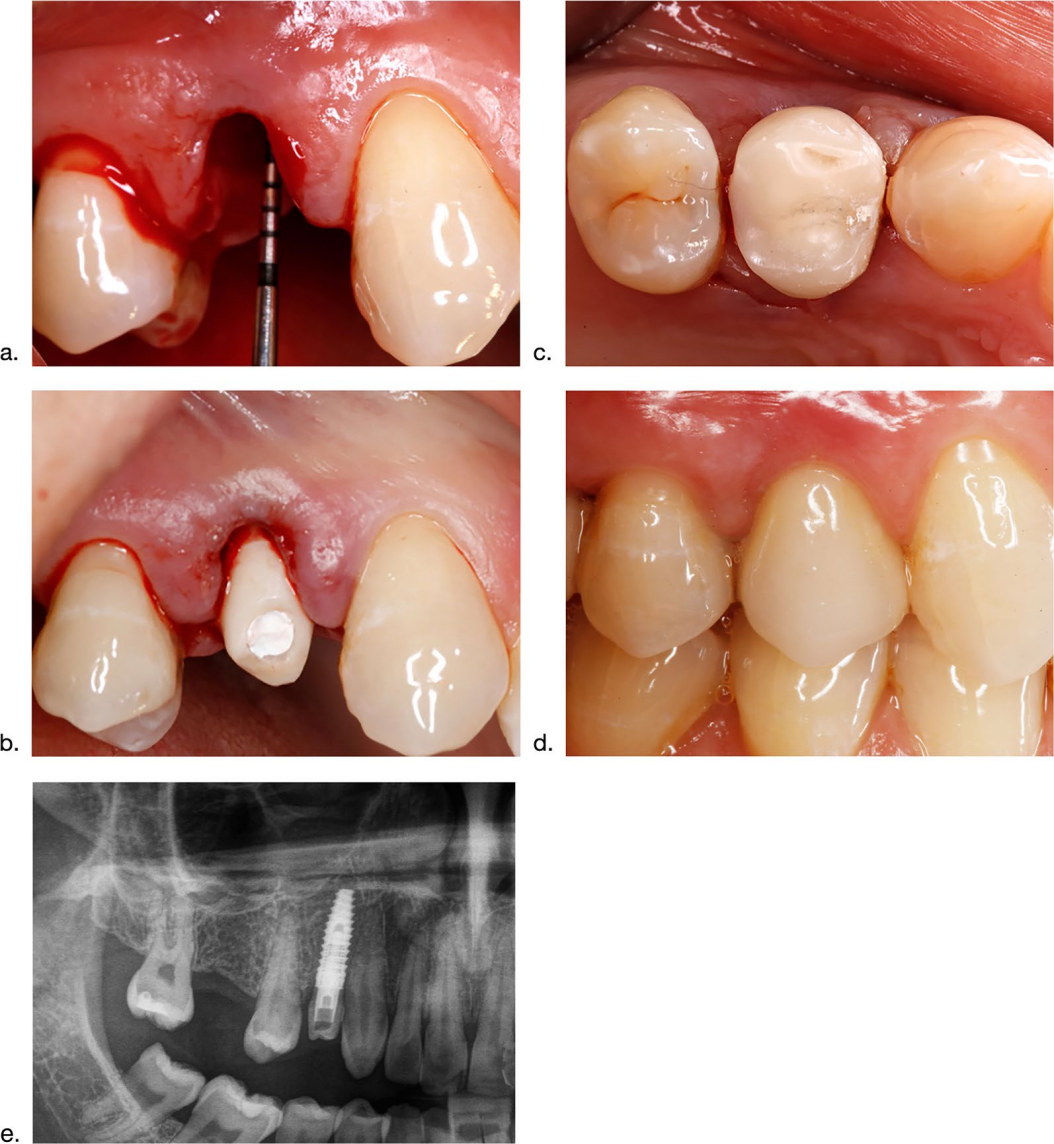
Surgical and prosthetic components of the protocol. **a** Clinical situation following tooth extraction region 014. **b** Immediate connection of the final abutment following implant placement. **c** Connection of the provisional crown in non-functional occlusion to seal the extraction socket and to protect and contain the bone graft material during the subsequent healing period of 3 months. **d** Clinical situation of the peri-implant soft tissues formed at the final cement- retained crown at 12 months. **e** Panoramic view following implant placement

An intraoral scan was completed intraoperatively (Cerec Omnicam; Dentsply SironaM, Bensheim, Germany) to assess the implant position (Camlog® ScanPosts for Sirona Scanbody) and the opposite jaws. The following four prosthetic components were fabricated within 2 h by the inhouse dental laboratory: (1) a definitive patient-specific hybrid abutment made of a titanium base abutment (Camlog®) and a bonded (Multilink Hybrid Abutment, Ivoclar-Vivadent, Ellwangen, Germany) mesostructure milled from a lithium disilicate blank (IPS e.max CAD Cerec/In Lab, Ivoclar-Vivadent), (2) a milled (Katana® Zirconia Block; Kuraray, Hattersheim, Germany) and rapidly sintered (CEREC SpeedFire Dental furnace, Dentsply Sirona) framework for the definitive crown, (3) a milled provisional crown (Vita CAD-Temp® monoColor, Vita, Bad Säckingen, Germany) without functional occlusion, and (4) a conventionally manufactured abutment replica for the extraoral cementation technique [12]. The definitive hybrid abutment was mounted with the recommended insertion torque. Subsequently, the ceramic framework was inserted and its 3D position recorded with an intraorally fabricated resin jig (Pro Temp™ 3 Garant, 3 M ESPE). The provisional crown was cemented with a temporary cement (Temp Bond; Kerr GmbH, Herzogenrath, Germany) using the abutment replica for the extraoral cementation technique. Patients were instructed to adhere to a soft diet for a period of 12 weeks. Within this period, the ceramic framework was remounted into the master cast using the transfer jig and veneered to a definitive crown with full occlusion (Cercon Ceram Kiss, Degudent, Hanau, Germany). After a healing period of 3 months, the definitive crown was cemented with a temporal cement (Temp Bond) (Fig. 1a–e).

### Assessment of primary and secondary outcomes

The following clinical measurements were recorded at final restoration (baseline), and at 6 and 12 months using a periodontal probe: (1) plaque index (PI) [13], (2) bleeding on probing (BOP), evaluated as present if bleeding was evident within 30 s after probing, or absent, if no bleeding was noticed within 30 s after probing, (3) probing depth (PD) measured from the mucosal margin to the bottom of the probeable pocket, (4) mucosal recession (MR) measured from the crown margin to the mucosal margin. All measurements were recorded at 6 aspects per implant: mesiovestibular (mb), midvestibular (b), distovestibular (db), mesiooral (mo), midoral (o), and distooral (do). All measurements were performed by two calibrated and experienced investigators (A.B., K.O.).

The esthetic outcome of the implant supported restoration was evaluated by two calibrated examiners (A.B., G.T.) using the pink esthetic score (PES) [14] and considering seven variables with a scoring from 2 (i.e. highest value) to 0 (i.e. lowest value). Accordingly, the highest possible score was 14.

The presence of peri-implant diseases at each implant site was assessed as follows: peri-implant mucositis: presence of BOP and/ or suppuration with or without increased PD; peri-implantitis: presence of BOP and/ or suppuration with increased PD and presence of radiographic bone loss (i.e. baseline to 6 and 12 months) [15]. According to the German X-ray ordinance based on 97/43/EURATOM directive and the radiation protection act based on the 103/2013 Euratom directive, routine two-dimensional radiographs for the assessment of marginal bone level changes at 6 and 12 months were not justified. Radiographs were just taken if clinically justified (i.e. in the presence of clinical signs suggesting the presence of peri-implantitis or mechanical/technical complications).

Implant survival was considered as the presence of the implant in situ at the 6- and 12 month follow-up examinations. Technical complications comprised all the events affecting the cemented crown [16]. Mechanical complications considered all the events affecting the integrity of the implant or of the abutment.

PROM’s were assessed by a questionnaire on the (1) prosthesis comfort, (2) prosthesis appearance, (3) chewing/(4) tasting ability, (5) prosthesis fit and (5) overall satisfaction with scores ranging from 1 (very satisfactory) to 5 (unsatisfactory).

### Postoperative care

Postoperative maintenance care included a supramucosal-/gingival professional implant/tooth cleaning and reinforcement of oral hygiene and provided at 6 and 12 months.

### Statistical analysis

The statistical analysis of the pseudonymised data sets was accomplished using a commercially available software program (IBM SPSS Statistics 27.0, IBM Corp., Armonk, NY, USA).

Mean values, standard deviations, medians, 95% confidence intervals (CI) and frequency distributions were calculated for all clinical parameters. The changes (d) in mean values from baseline to 6 and 12 months were examined with the Shapiro–Wilk test. In a next step, within group comparisons of dBOP, dPD, dPI and dPES values were accomplished using the Wilcoxon signed-rank test. Linear regression analyses were used to assess the relationship between defect width/ length and vestibular BOP values at 6 and 12 months. The alpha error was set at 0.05.

## Results

The extraction sockets were commonly associated with minor to moderate buccal dehiscence- type defects. The corresponding mean width and length amounted to 1.33 ± 2.69 mm (median: 0.0; 95% CI − 0.74; 3.4 mm) and 3.11 ± 6.19 mm (median: 0.0; 95% CI − 1.65; 7.87 mm), respectively. An adequate primary implant stability (i.e. insertion torque > 35 Ncm) was obtained at all but one sites. In the latter case, the implant site was submerged employing a free gingival punch from the palate. Accordingly, a total of *n* = 20 were finally evaluated.

### Implant survival

At 6 and 12 months, all implants were in place and exhibited an absence of clinical mobility, thus resulting in survival rates of 100%, respectively.

### Clinical measurements

Mean and median BOP, PD, MR, PI, and PES values measured at 6 and 12 months are summarized in Table 3, 4, 5.

**Table 3:** Clinical parameters measured at baseline (i.e. final restoration) (*n* = 20 patients)

	BOP	PD	MR	PI	PES
Mean	9.17	2.86	0.01	0.00	12.6
SD	15.74	0.83	0.05	0.00	1.34
Median	0.00	2.83	0.00	0.00	13.00
95% CI	1.8;16.5	2.47;3.25	− 0.01;0.03	0.00;0.00	11.97;13.22

**Table 4:** Clinical parameters measured at 6 months (*n* = 20 patients)

	BOP	PD	MR	PI	PES
Mean	10.00	2.70	0.01	0.01	12.9
SD	16.57	0.50	0.05	0.05	1.14
Median	0.00	2.75	0.00	0.00	13.00
95% CI	2.2;17.7	2.47;2.94	− 0.01;0.03	0.00;0.04	12.36;13.43

**Table 5:** Clinical parameters measured at 12 months (*n* = 20 patients)

	BOP	PD	MR	PI	PES
Mean	11.67	2.60	0.01	0.14	13.2
SD	20.30	0.49	0.05	0.33	0.84
Median	0.00	2.66	0.00	0.00	13.50
95% CI	2.1;21.1	2.37;2.82	0.00;0.04	− 0.01;0.29	12.80;13.59

Throughout the follow-up period of 12 months, all patients investigated exhibited a good level of plaque control, as indicated by median PI scores of 0.00 at respective implant sites (Tables 3, 4, 5). At 6 and 12 months, mean BOP scores were commonly considered as low with only minor changes to baseline amounting to 0.8 ± 22.6% and 2.5 ± 28.2%, respectively (*p* = 0.858, *p* = 0.799, Wilcoxon signed-rank test) (Tables 3, 4, 5, 6 and 7). Mean PD scores slightly decreased by 0.15 ± 0.66 mm and 0.26 ± 0.73 mm at 6 and 12 months, respectively (*p* = 0.276, *p* = 0.102, Wilcoxon signed-rank test) (Tables 3, 4, 5, 6 and 7). In all patients investigated, mean MR values remained unchanged at 6 and 12 months, respectively (*p* = 1.00, *p* = 0.655, Wilcoxon signed-rank test) (Tables 3, 4, 5, 6 and 7). The mean PES values amounted to 12.9 ± 1.14 and 13.2 ± 0.84 at 6- and 12 months, respectively. The resulting changes of 0.6 ± 1.0 noted from baseline to 12 months reached statistical significance (*p* = 0.032, Wilcoxon signed-rank test) (Tables 3, 4, 5, 6 and 7).

**Table 6:** Changes (d) in clinical parameters between baseline and 6 months (*n* = 20 patients)

	dBOP	dPD	dMR	dPI	dPES
Mean	0.8 ± 22.6	− 0.15 ± 0.66	0.0 ± 0.0	0.01 ± 0.05	0.3 ± 1.1
Median	0.0	− 0.08	0.0	0.00	0.0
95% CI	− 9.75; 11.41	− 0.46; 0.15	0.0; 0.0	0.00; 0.04	− 0.23; 0.83
*p* value	0.858	0.276	1.00	0.157	0.369

Within group comparison V8–V9: ^*^ Wilcoxon signed-rank test

**Table 7:** Changes (d) in clinical parameters between baseline and 12 Months (*n* = 20 patients)

	dBOP	dPD	dMR	dPI	dPES
Mean	2.5 ± 28.2	− 0.26 ± 0.73	0.0 ± 0.4	0.14 ± 0.33	0.60 ± 1.0
Median	0.0	− 0.16	0.0	0.00	0.5
95% CI	− 10.72; 15.72	− 0.61; 0.07	− 0.01; 0.02	− 0.01; 0.29	0.09; 1.10
*p* value	0.799	0.102	0.655	0.066	0.032^*^

Within group comparison V8–V9: ^*^ Wilcoxon signed-rank test

### Incidence of peri-implant diseases

The frequency distribution of peri-implant disease at 6 and 12 months are summarized in Table 8. According to the given case definitions, the incidence of peri-implant mucositis and peri-implantitis at both patient- and implant levels amounted to 30.00% and 0.0% at 6 months and to 40.00% and 0.0%, respectively.

**Table 8:** Incidence of peri-implant diseases at 6 and 12 months

		Diagnosis			Total
		0	1	2	
3 Months	Count	14	6	0	20
	%	70.0%	30.0%	0.0%	100.0%
6 Months	Count	12	8	0	20
	%	60.0%	40.0%	0.0%	100.0%

Diagnosis: 0: peri-implant health; 1: peri-implant mucositis; 2: peri-implantitis

### Regression analysis

The linear regression analysis failed to reveal any significant correlations between either defect width or defect length and mean vestibular BOP values at 6 (Coef: 0.221, *R*^2^ = 0.049, *p* = 0.348; Coef: 0.221, *R*^2^ = 0.049, *p* = 0.348) and 12 months (Coef: 0.034, *R*^2^ = 0.001, *p* = 0.887; Coef: 0.01, *R*^2^ = 0.000, *p* = 0.966), respectively.

## Incidence of technical and mechanical complication

Throughout the observation period of 12 months, none of the implant supported restorations was associated with any technical or mechanical complications.

### PROM’s

**Fig. 2:**
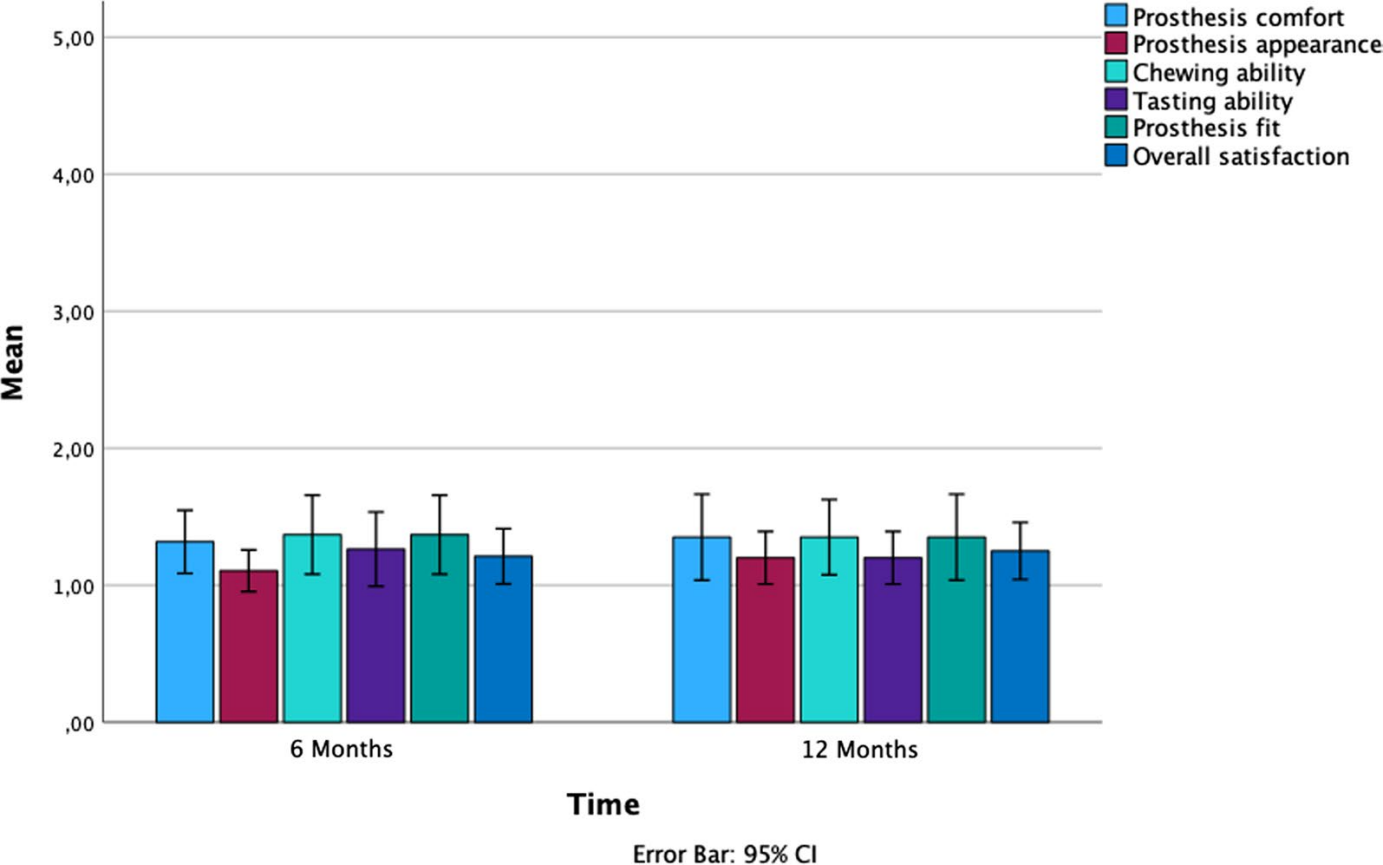
Depiction of the PROM’s assessed at 6 and 12 months with scores ranging from 1 (very satisfactory) to 5 (unsatisfactory)

The mean values for the evaluated questionary items 1–6 at 6 months amounted to 1.32 ± 0.47 (min. 1; max. 2) (i.e. prosthesis comfort), 1.11 ± 0.31 (min. 1; max. 2) (i.e. prosthesis appearance), 1.37 ± 0.59 (min. 1; max. 3) (i.e. chewing ability), 1.26 ± 0.56 (min. 1; max. 3) (i.e. tasting ability), 1.37 ± 0.59 (min. 1; max. 3) (i.e. prosthesis fit), and 1.21 ± 0.41 (min. 1; max. 2) (i.e. overall satisfaction. At 12 months, these values amounted to 1.36 ± 0.68 (min. 1; max. 3), 1.21 ± 0.41 (min. 1; max. 2), 1.31 ± 0.58 (min. 1; max. 3), 1.21 ± 0.41 (min. 1; max. 2), 1.36 ± 0.68 (min. 1; max. 3), and 1.26 ± 0.45 (min. 1; max. 2), respectively (Fig. 2).

## Discussion

The present prospective observational study aimed at evaluating the survival and success of immediately placed and restored PL implants for single-tooth replacements in the esthetic zone.

Within the limitations of a short-term follow-up period of 12 months, the survival rate amounted to 100% and all implant sites investigated revealed non-significant changes in mean BOP (2.5 ± 28.2%), PD (-0.26 ± 0.73 mm), and MR (0.0 ± 0.4 mm) values when compared with the respective baseline values. This was commonly associated with high PES values amounting to 12.9 ± 1.14 and 13.2 ± 0.84 at 6 and 12 months, respectively. Furthermore, the noted absence of any technical or mechanical complications also contributed to the commonly high patient satisfaction.

In this context, it must be emphasized that the present observational study had a proof-of-principle character, since a sample size calculation was not feasible due to a lack of appropriate reference data in the literature. While the sample size may therefore not have the statistical power to rule out significant within group changes at 6 and 12 months, the very minor differences to baseline as noted for all clinical outcomes do not appear to be of clinical relevance. Nevertheless, the presented data should serve as basis for an adequate sample size calculation in future studies.

Basically, the present survival rates and clinical outcomes noted for immediately placed and restored PL implants appear to be within the range of previous short-term follow-up studies employing similar restoration concepts for single tooth replacements in the anterior maxilla (i.e. central incisor to premolar region) [17–19]. In particular, at 12 months, Cooper et al. [17] reported on a 94.8% survival rate of two-part implants exhibiting a non-progressive thread design with the majority of implant sites (83.7%) showing a gain in marginal soft tissue levels. Likewise, Raes et al. [20] reported on one early implant loss with a total of 16 patients completing the short-term follow-up of 12 months. The lengths of the same implant type that was used in the latter study ranged between 13 and 17 mm. Mean PES values amounted to 10.33 ± 2.29, thus merely indicating an acceptable esthetic result for the vast majority of implant sites. Nevertheless, the midfacial soft tissue levels also remained stable over time [20].

For a two-part implant exhibiting a progressive- thread design, the survival rate at 12 months also amounted to 100% [3]. Mean bleeding scores and PD values (buccal aspect) changed from 0.60 ± 0.60 and 2.65 ± 1.42 mm at baseline to 0.25 ± 0.44 and 3.05 ± 0.83 mm at 12 months, respectively. The mean PES values changed from 7.80 ± 1.66 to 7.50 ± 1.59 at 12 months, thus resulting in acceptable clinical outcomes in about 94% of the patients investigated [3].

Potential differences noted between the aforementioned studies and the present analysis might be due to variations in either the surgical (e.g. integrity of the extraction socket, implant types, required insertion torques, gap filling, concomitant soft tissue grafting) or restorative (e.g. provisional vs. full loading) components of the respective protocols. In this context, it is important to emphasize that the present study adhered to the clinical recommendations for immediate implant placement as elaborated during the XV European Workshop in Periodontology [1]. This particularly applied to the exclusion of severely damaged (i.e. “more than 50% loss of one or more walls”) extraction sockets as well as implantation of defect grafting as integral component of the surgical procedure.

In this context, it must be emphasized that non-intact buccal bone walls may be associated with increased PD- and decreased PES values [1], thus underlining the importance of a proper case selection. However, when further analyzing the present regression analysis, it was noted that the sizes (i.e. width and length) of the minor to moderate buccal dehiscence- type defects had no significant influence on changes in BOP values and subsequently the incidence of peri-implant diseases.

The prosthetic protocol defined for the present study also followed an established workflow as reported previously [21]. One distinct component of the protocol is the immediate connection of the final abutment at implant placement to allow for a “one abutment-one time concept” [22], which may have contributed to the stable MR values and high PES scores noted at 6 and 12 months. In fact, repeated abutment manipulations were proven to be associated with a higher occurrence of biological complications, a higher marginal bone loss as well as greater buccal recessions when compared with the control group (i.e. no abutment manipulation) [22, 23].

The stability of these promising outcomes, however, needs to be verified on the medium- and long- term. In addition, future studies should account for the potential drawbacks associated with the present analysis, such as the lack of a sample size calculation, the limited number of participants, as well as the lack of a control group.

## Conclusions

In conclusion and within its limitations, the presented immediacy protocol was associated with high survival and success rates on the short-term.

## Data Availability

Data are available on request due to privacy or other restrictions.

## Abbreviations

BOP: Bleeding on probing
PD: Probing depth
PI: Plaque index
MR: Mucosal recession
PES: Pink esthetic score
PL: Progressive-type two-piece implant
PROM: Patient-reported outcome measure
